# Deep learning for standardized, MRI-based quantification of subcutaneous and subfascial tissue volume for patients with lipedema and lymphedema

**DOI:** 10.1007/s00330-022-09047-0

**Published:** 2022-08-17

**Authors:** Sebastian Nowak, Andreas Henkel, Maike Theis, Julian Luetkens, Sergej Geiger, Alois M. Sprinkart, Claus C. Pieper, Ulrike I. Attenberger

**Affiliations:** Department of Diagnostic and Interventional Radiology, Quantitative Imaging Lab Bonn (QILaB), University Hospital Bonn, Venusberg-Campus 1, 53127 Bonn, Germany

**Keywords:** Deep learning, Magnetic resonance imaging, Lymphography, Leg, Subcutaneous tissue

## Abstract

**Objectives:**

To contribute to a more in-depth assessment of shape, volume, and asymmetry of the lower extremities in patients with lipedema or lymphedema utilizing volume information from MR imaging.

**Methods:**

A deep learning (DL) pipeline was developed including (i) localization of anatomical landmarks (femoral heads, symphysis, knees, ankles) and (ii) quality-assured tissue segmentation to enable standardized quantification of subcutaneous (SCT) and subfascial tissue (SFT) volumes. The retrospectively derived dataset for method development consisted of 45 patients (42 female, 44.2 ± 14.8 years) who underwent clinical 3D DIXON MR-lymphangiography examinations of the lower extremities. Five-fold cross-validated training was performed on 16,573 axial slices from 40 patients and testing on 2187 axial slices from 5 patients. For landmark detection, two EfficientNet-B1 convolutional neural networks (CNNs) were applied in an ensemble. One determines the relative foot-head position of each axial slice with respect to the landmarks by regression, the other identifies all landmarks in coronal reconstructed slices using keypoint detection. After landmark detection, segmentation of SCT and SFT was performed on axial slices employing a U-Net architecture with EfficientNet-B1 as encoder. Finally, the determined landmarks were used for standardized analysis and visualization of tissue volume, distribution, and symmetry, independent of leg length, slice thickness, and patient position.

**Results:**

Excellent test results were observed for landmark detection (z-deviation = 4.5 ± 3.1 mm) and segmentation (Dice score: SCT = 0.989 ± 0.004, SFT = 0.994 ± 0.002).

**Conclusions:**

The proposed DL pipeline allows for standardized analysis of tissue volume and distribution and may assist in diagnosis of lipedema and lymphedema or monitoring of conservative and surgical treatments.

**Key Points:**

• *Efficient use of volume information that MRI inherently provides can be extracted automatically by deep learning and enables in-depth assessment of tissue volumes in lipedema and lymphedema*.

• *The deep learning pipeline consisting of body part regression, keypoint detection, and quality-assured tissue segmentation provides detailed information about the volume, distribution, and asymmetry of lower extremity tissues, independent of leg length, slice thickness, and patient position*.

**Supplementary Information:**

The online version contains supplementary material available at 10.1007/s00330-022-09047-0.

## Introduction

A chronic increase in leg circumference—either uni- or bilateral—can be caused by a range of pathological conditions: apart from venous disease and obesity, lymphedema and lipedema are recognized as major causes of increased extremity circumference [1, 2]. Lymphedema is characterized by soft tissue swelling caused by impaired lymphatic drainage leading to an accumulation of interstitial fluid. Through inflammatory reactions, a progressing deposition of subcutaneous fat, tissue fibrosis, and ultimately skin changes can be observed [2]. Lipedema is a disorder characterized by adipose tissue accumulation in the extremities and predominantly affects females. Patients typically present with a disproportionate distribution of body fat on the extremities despite a slender upper body and further symptoms such as fatigue and hyperalgesia. Additionally, lymphedema may develop in the affected patients [3, 4]. The pathophysiology of lipedema is so far poorly understood [1, 4, 5]. In patients suffering from either lipedema or lymphedema, both mechanic impairments—that can cause secondary arthritis or interfere with normal walking—and emotional disorders—resulting from an appearance that does not conform to today’s ideal of beauty—can result in impaired quality of life [1].

Traditionally, the diagnosis of lipedema and lymphedema is made by clinical examination with evaluation of leg circumference, pitting edema, pain, typical clinical signs (e.g., Stemmer’s sign), standardized anthropometric measurements (e.g., body weight, body mass index, waist-to-hip ratio, waist-to-height ratio), and patient history [1, 2, 4]. Especially since the introduction of microsurgical treatment options for lymphedema, a more in-depth evaluation of the affected legs by clinical MRI—e.g., as MR-lymphangiography (MRL)—has been introduced at specialized centers for treatment planning and therapy monitoring [6, 7]. In this respect, MRI is increasingly employed in the diagnosis, staging assessment, and follow-up of both lipedema and lymphedema and especially multi-echo T1-weighted images (e.g., using the DIXON technique) have been demonstrated to be useful for anatomical evaluation [8–11]. As simple anthropometric measures do not allow for separate assessment of subfascial and subcutaneous tissue and do not provide information on the volume distribution of these tissues along the entire extremities, it is therefore a logical step to leverage available imaging for precise volume assessment of these different compartments.

In recent years, DL methods have shown their potential to automate the quantification of tissue volumes in medical image analysis [12–15]. Therefore, DL could also provide a useful tool for automated imaging-based assessment of tissue volume in patients with suspected lipedema or lymphedema.

For a clinical application of artificial intelligence–based systems, it is important that the autonomous procedure has quality control mechanisms that are able to warn the treating physician in case of potentially limited validity of the measurement [14]. Quality control is not only important for evaluating individual examinations, but it can also be used to monitor the performance of the system over the time of deployment. The hardware requirements and the time required for inference are other aspects that affect the economics and accessibility of the automated systems, making a comparison of performance and efficiency of different DL models of interest.

Therefore, it was the aim of this study to develop a DL pipeline that allows to automatically extract precise normalized information of tissue volume, distribution, and symmetry from available MRI of the legs of patients with lipedema or lymphedema for standardized quantification of subcutaneous tissue (SCT) and subfascial tissue (SFT), while investigating the performance and efficiency of different architectures.

## Material and methods

### Dataset

This retrospective study was approved by the institutional review board with a waiver for written informed consent for data analysis. Consecutive patients who underwent clinical MRL examinations of the lower extremities between April 2016 and May 2017 were included into the study when they had either clinically diagnosed lymphedema (primary or secondary) or lipedema of the lower extremities. The indication for imaging was treatment planning (e.g., of lympho-venous anastomoses) in all patients. 3D DIXON MRL (slice thickness 5 mm, spacing between overlapping reconstructed slices 2.5 mm, in-plane resolution 1 mm) was performed as part of the pre-therapeutic diagnostic work-up on a 1.5-T MR system (Ingenia; Philips Healthcare) to assess gross and lymphatic anatomy as well as presence and extent of lymphatic run-off impairment. Clinical diagnosis was made by the referring experienced lymphologists based on the national guidelines for lymphedema and lipedema [4, 17].

Overall, 45 patients (42 female, mean age 44.2 ± 14.8 years) were examined during the selected time period and were included into the study. Of 45 patients, 36 (80%) suffered from lymphedema (13 primary, 23 secondary) and 9/45 (20%) from lipedema, with all men having secondary lymphedema and receiving MRL for treatment planning of lympho-venous anastomoses. Exclusively, DIXON water images were used for method development. In total, the dataset consisted of 18,760 slices in axial orientation. Data were randomly split into a training set for five-fold cross-validation of 40 (38 female, mean age 45.0 ± 15.5 years) cases and a hold-out test of 5 cases (4 female, mean age 37.4 ± 4.5 years) set. Detailed information on imaging parameters and image pre-processing prior to training can be found in Supplement S1.

The ground truth generation for the landmark detection was performed manually using Slicer 3D [18]. For the tissue segmentation, semi-manual tools and AI-assisted annotation were applied by a research assistant (S.N. with 3 years of experience in medical image segmentation). All annotations were finally approved by a board-certified radiologist (C.C.P. with 10 years of experience in lymphatic imaging). Further information on annotation can be found in Supplement S2.

The DL pipeline was finally also applied to four different use cases of routine clinical practice to demonstrate the clinical utility of the presented method for assessing tissue volume, distribution, and symmetry of the lower extremities and for monitoring of conservative or surgical treatment.

### Method development for leg normalization

**Fig. 1:**
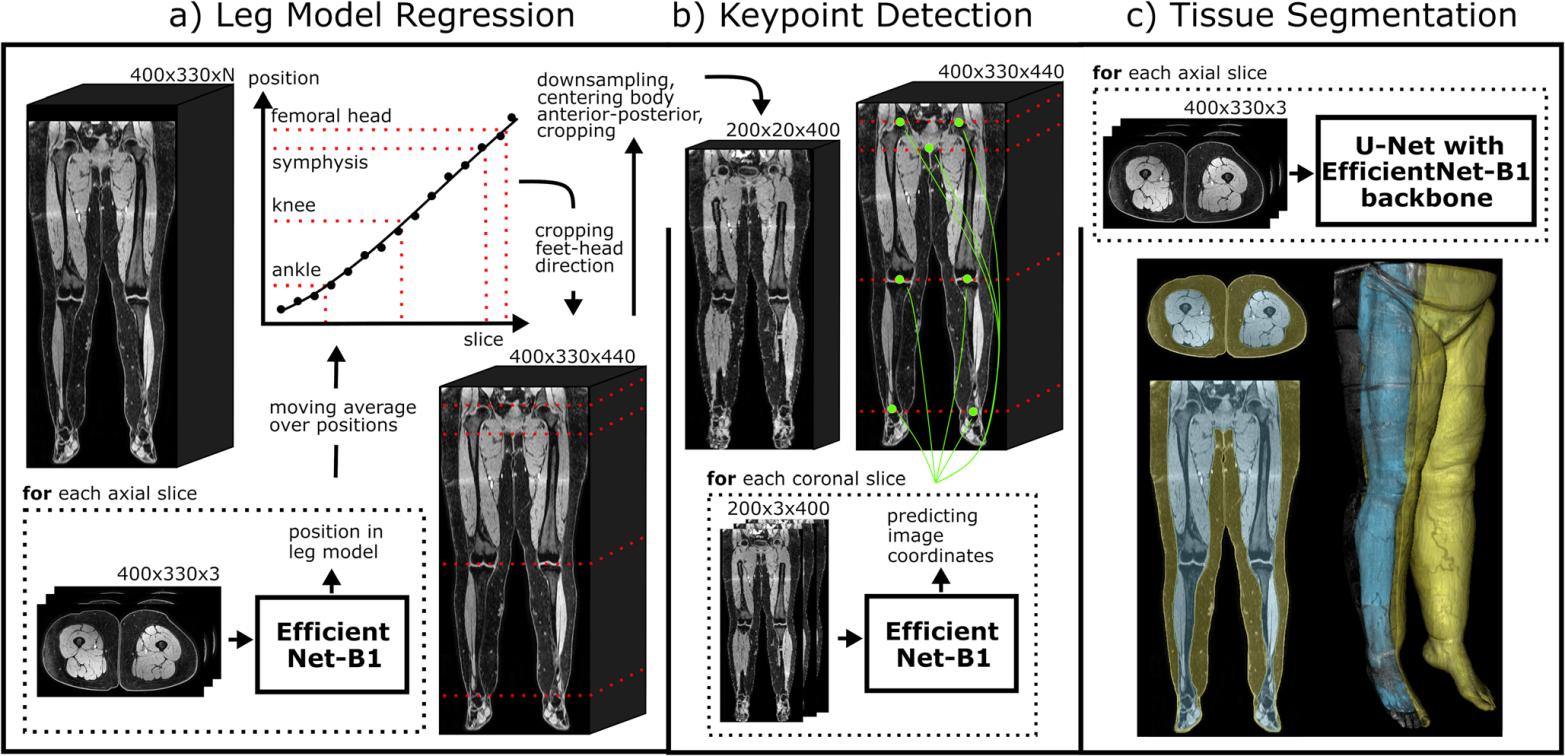
Overview of the DL pipeline. (**a**) First, the 3D MRI scan is analyzed in axial slices by a 2.5D EfficientNet-B1 to identify the relative foot-head position of each slice with respect to a leg model consisting of ankles, knees, symphysis, and femoral heads. Afterwards, the image dataset is automatically cropped to the legs. (**b**) To increase the accuracy of the leg normalization, all landmarks are predicted by another 2.5D EfficientNet-B1 in coronal slices of a down-sampled cropped image using keypoint detection, where the lower limbs were centered slice-by-slice in anterior-posterior direction to the image center. (**c**) Then, a 2.5D U-Net with EfficientNet-B1 as backbone is used for segmentation of subcutaneous adipose tissue and subfascial tissue volume in axial slices. Finally, the identified landmarks and tissue volumes are combined to allow standardized quantification of the tissues (see Figs. 3 and 4)

Figure 1 shows an overview of the developed pipeline consisting of two landmark detection methods and a quality-controlled tissue segmentation method.

#### (i) Leg model regression

In the first landmark detection method, a 2.5D CNN encoder determines the relative foot-head position of each axial slice within a standardized leg model by regression. To create the standardized leg model, the mean distances between the manually defined landmarks were determined for the entire dataset (ankle-knee: 95.0 ± 6.7 cm, knee-symphysis: 99.1 ± 6.1 cm, symphysis-femoral head: 16.7 ± 1.8 cm). The distances between the landmarks were normalized to the mean distance between ankles and knees, resulting in the relative positions −1, 0, 1.045, and 1.220 for ankles, knees, symphysis, and femoral heads within the leg model. The position values of the slices between the landmarks were linearly interpolated. Two numbers were then assigned to each axial slice, which corresponded to the relative position of that slice in the leg model for the left and for the right leg. Subsequently, image areas superior to the femoral heads were excluded from further analyses.

#### (ii) Keypoint detection

In the second landmark detection method, an additional 2.5D CNN encoder detects the image coordinates of the landmarks in coronal reconstructed slices using keypoint detection. To create the coronal reconstructed slices, the cropped images are down-sampled to an isotropic resolution of 2.5 mm. Then, slice by slice, the center of mass of the body mask was shifted in the anterior-posterior direction to the center of the image. Subsequently, the image matrix was cropped at a distance of 25 mm anterior and 25 mm posterior from the center of the image. This area contained all landmarks.

For processing 3D information, 2.5D CNN encoders with three axial slices spaced 5 mm apart between each slice were used as input channels for all CNNs used in the current study. Also, different versions of a modern implementation of the established ResNet (ResNet18, ResNet34, ResNet50), as well as different versions of the recently introduced EfficientNets (B0, B1, B2, B3, B4), were implemented for both landmark detection methods [19, 20]. The most appropriate model was selected based on validation performance and model efficiency in terms of the number of trainable parameters and floating point operations required. For training the landmark detection methods, five-fold cross-validation was used and testing was performed with an ensemble of the cross-validated models.

### Method development for tissue segmentation

#### Model

2.5D models with a U-Net-like architecture were investigated to segment the subcutaneous adipose tissue in the 3D MRI scans. Again, different versions of ResNet (ResNet18, ResNet34) and EfficientNet (B0, B1, B2, B3, B4) encoders were implemented for the U-Net model [19, 20]. The application of ResNet as an encoder of a U-Net has already been demonstrated to be able to segment the liver with high precision in MRI [21]. In addition, a CDFNet was trained, which was recently presented for abdominal adipose tissue segmentation in DIXON MRI images and computed tomography [13, 14]. Again, the most appropriate model was selected based on validation performance and model efficiency in terms of the number of trainable parameters and floating point operations required. Subsequently, the chosen 2.5D network architecture was also trained to perform segmentation on sagittal and coronal slices to investigate if a multi-view approach is beneficial for the current segmentation task [13].

As with the landmark detection methods, five-fold cross-validation was used for training. Testing was performed with an ensemble of the cross-validated models. Detailed information on the network architectures used as well as the hyperparameters used for training the tissue segmentation and landmark detection methods can be found in Appendix S3 and S4.

#### Quality control

For automatic assessment of segmentation quality, the entropy of the probability map of the segmentation models was used as a metric to predict the prediction uncertainty as in terms of the Dice score as proposed in previous studies [14, 16].

In the current study, two linear regression models were trained. One based on the entropy of the entire probability map of the 3D segmentation, as proposed in the original work, and another which considers the entropy slice by slice [16]. By this, it should be investigated whether this allows a local evaluation of the quality and thus represents a beneficial extension of the 3D approach. Only slices between the ankles and femoral heads with segmentations larger than 10% (12.7 cm^2^) of the image section were considered. The linear regression models were trained with the predicted segmentations of all validation cases of the cross-validated tissue segmentation method and tested on the hold-out test set. Pearson correlation (*r*) coefficients were calculated with SciPy 1.6.3 [22].

## Results

### Leg normalization

EfficientNet-B1 was chosen as the most suitable model for both landmark detection methods used for leg normalization as it showed excellent performance in the five-fold cross-validation while having the least number of trainable parameters and floating point operations, resulting in a prediction time per patient of 2.7 s for the first method and 0.1 s for the second method on an NVIDIA Titan RTX graphics processing unit (GPU). Low mean deviations (Δ*z*) between the predictions of the validation cases of the cross-validated landmark detection models and the manually defined ground truth were observed for the leg model regression (Δ*z* = 6.6 ± 2.7 mm) and for the keypoint detection (Δ*z* = 6.6 ± 3.2 mm). The mean sex-specific deviations were Δ*z*-female = 6.4 ± 2.6 mm, Δ*z*-male = 10.0 ± 4.4 mm for the leg model regression and Δ*z*-female = 6.6 ± 3.2 mm, Δ*z*-male = 7.9 ± 4.0 mm for the keypoint detection.

The ensemble of all cross-validated models showed also low mean deviations on the hold-out test set (leg model regression: Δ*z* = 5.6 ± 5.6 mm; keypoint detection: Δ*z* = 6.9 ± 4.4 mm). Considering an acceptable deviation of up to 10 mm in the test set, 85.7% of the landmarks detected by leg model regression and 74.3% of the landmarks detected by keypoint detection were correct. Using the predictions in an ensemble, the performance increased to Δ*z* = 4.5 ± 3.1 (100% < 10 mm).

### Tissue segmentation

EfficientNet-B1 was also chosen as the most suitable model for tissue segmentation as it showed again excellent segmentation performance on axial slices in the five-fold cross-validation with mean Dice scores of 0.982 ± 0.007 for segmenting SCT and of 0.989 ± 0.003 for segmenting SFT. The mean prediction time per patient was 8 s on an NVIDIA RTX 3090 GPU. Dice scores were consistently above 0.95 for both genders (Dice score female: SCT = 0.983 ± 0.007, SFT = 0.989 ± 0.003; Dice score male: SCT = 0.967 ± 0.005, SFT = 0.984 ± 0.003). Combining the predictions of three multi-view models, each segmenting on either axial, coronal, and sagittal slices, did not improve the already very high segmentation performance (Dice SCT: 0.980 ± 0.008; Dice SFT: 0.987 ± 0.004).

An ensemble of the five-fold cross-validated EfficientNet-B1 models applied to axial slices achieved also excellent Dice scores on the test set (Dice SCT: 0.989 ± 0.004; Dice SFT: 0.994 ± 0.002).

Detailed information and illustrations on the model selection for tissue segmentation and landmark detection can be found in Supplement S5.

#### Quality control

**Fig. 2:**
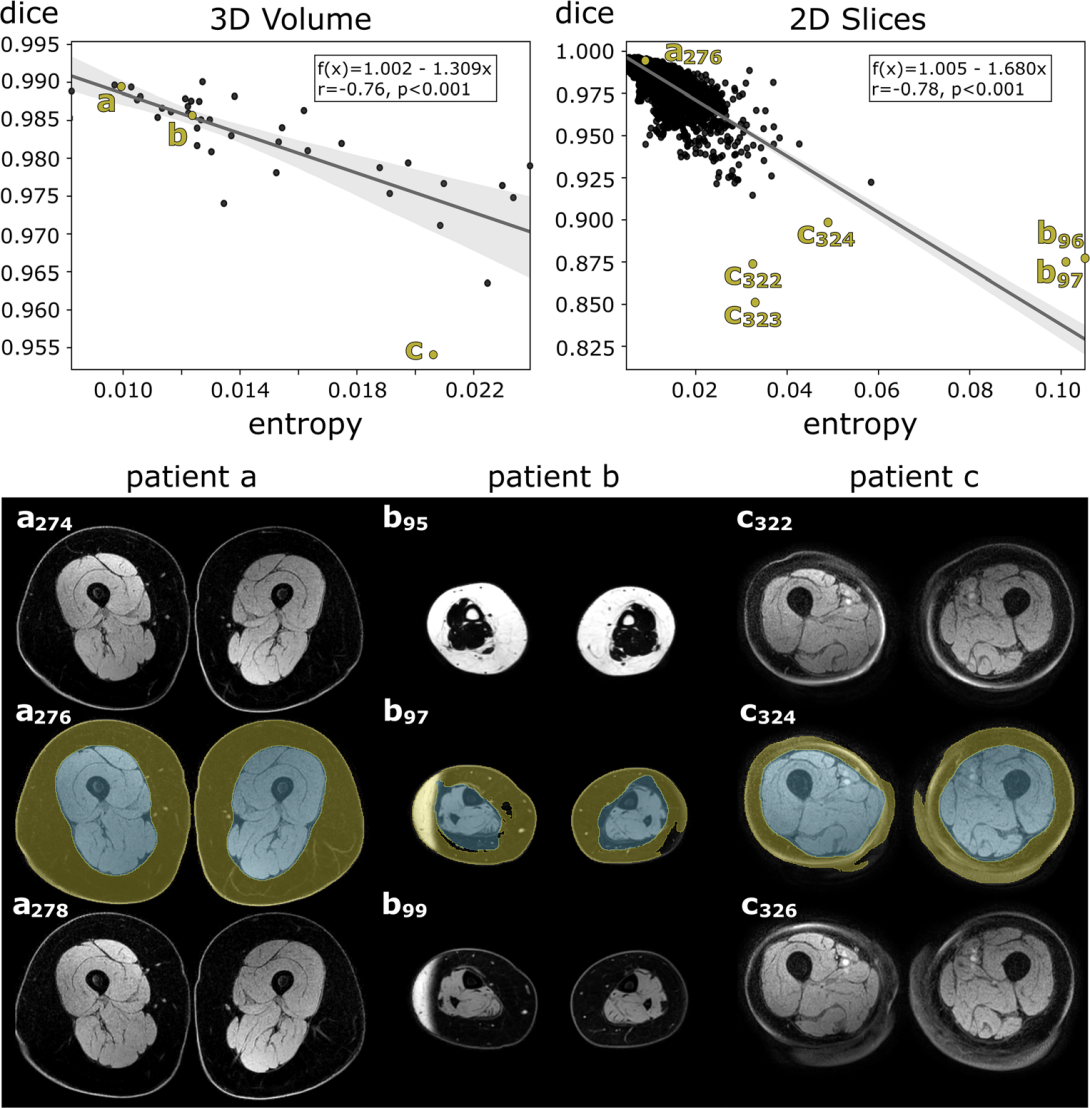
The two linear regression models developed for quality control of the tissue segmentation convolutional neural network (CNN) are shown in the upper section of the figure. These are used for predicting the segmentation quality of the subcutaneous tissue class in terms of the Dice score. The first regression model was based on the entropy of the entire probability map of the 3D segmentation (top left). A second regression model was developed to predict segmentation quality slice by slice (top right). Gray areas represent 95% confidence intervals. Pearson correlation coefficient (*r*) along with the two-tailed p-value is given in the boxes. The lower section of the figure shows the 3 channel inputs of the 2.5D segmentation CNN for three patients (a, b, c), respectively, whose entropy of probability map and Dice scores are highlighted in the plot above. The digits represent the slice numbers. Excellent overall segmentation quality with high Dice scores and low entropy was observed for the majority of the entire 3D volumes and 2D slices (c.f. patient a). The slice-wise prediction of the Dice score allows to additionally capture local effects on segmentation quality caused, e.g., by water-fat swap (as seen in patient b) or partial volume artefacts (as seen in patient c). For patients b and c, adjacent artifact-affected slices, which also had low predicted Dice scores, are also highlighted in the plot above

Both linear regression models (based on 3D volumes and 2D slices) demonstrated a high correlation between the entropy of the subcutaneous tissue segmentation probability map and the segmentation quality represented by the Dice score (3D volumes: SCT *r* = −0.76 *p* < 0.001, SFT *r* = −0.75 *p* < 0.001; 2D slices: SCT *r* = −0.78 *p* < 0.001, SFT *r* = −0.76 *p* < 0.001). Low mean deviation between predicted and actual Dice score were observed when applying the models to the hold-out test set (3D volumes: ΔDice SCT: 0.003 ± 0.002; ΔDice SFT: 0.001 ± 0.001; 2D slices: ΔDice SCT: 0.003 ± 0.002; ΔDice SFT: 0.002 ± 0.003). Figure 2 shows the two regression models and also illustrates the application of the models for automatic identification of cases with lower segmentation quality.

### Use cases

**Fig. 3:**
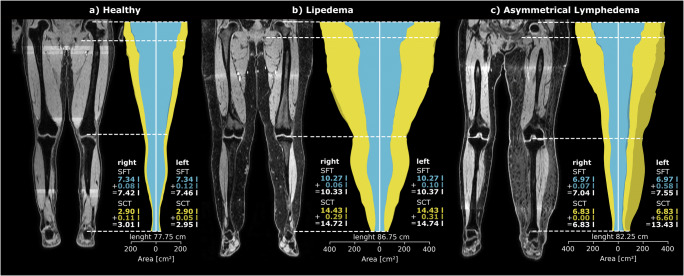
Use cases for assessment of volume, distribution, and symmetry utilizing volume information from MRI. On the left (**a**) is a patient (female, 45 years old) without swelling of the lower extremities, in the middle (**b**) is a patient with lipedema (female, 46 years old), and on the right (**c**) is a patient with asymmetric left secondary lymphedema (female, 66 years old). Cumulative axial tissue areas are displayed per slice for each patient, with the distribution of the subfascial tissue (SFT) shown in blue and of the subcutaneous tissue (SCT) in yellow separated for the left and right leg between the femoral heads and the ankles. The detected landmarks are indicated by dotted lines. In order to highlight the differences in tissue volume between the two legs, asymmetric tissue portions are shown in darker blue for SFT and darker yellow for SCT. This is particularly apparent in the illustration of the patient with asymmetrical lymphedema (**c**). Next to the right and left leg, the tissue volumes are indicated in liters with corresponding colored font, and the total volume of SFT and SCT is indicated with white font

**Fig. 4:**
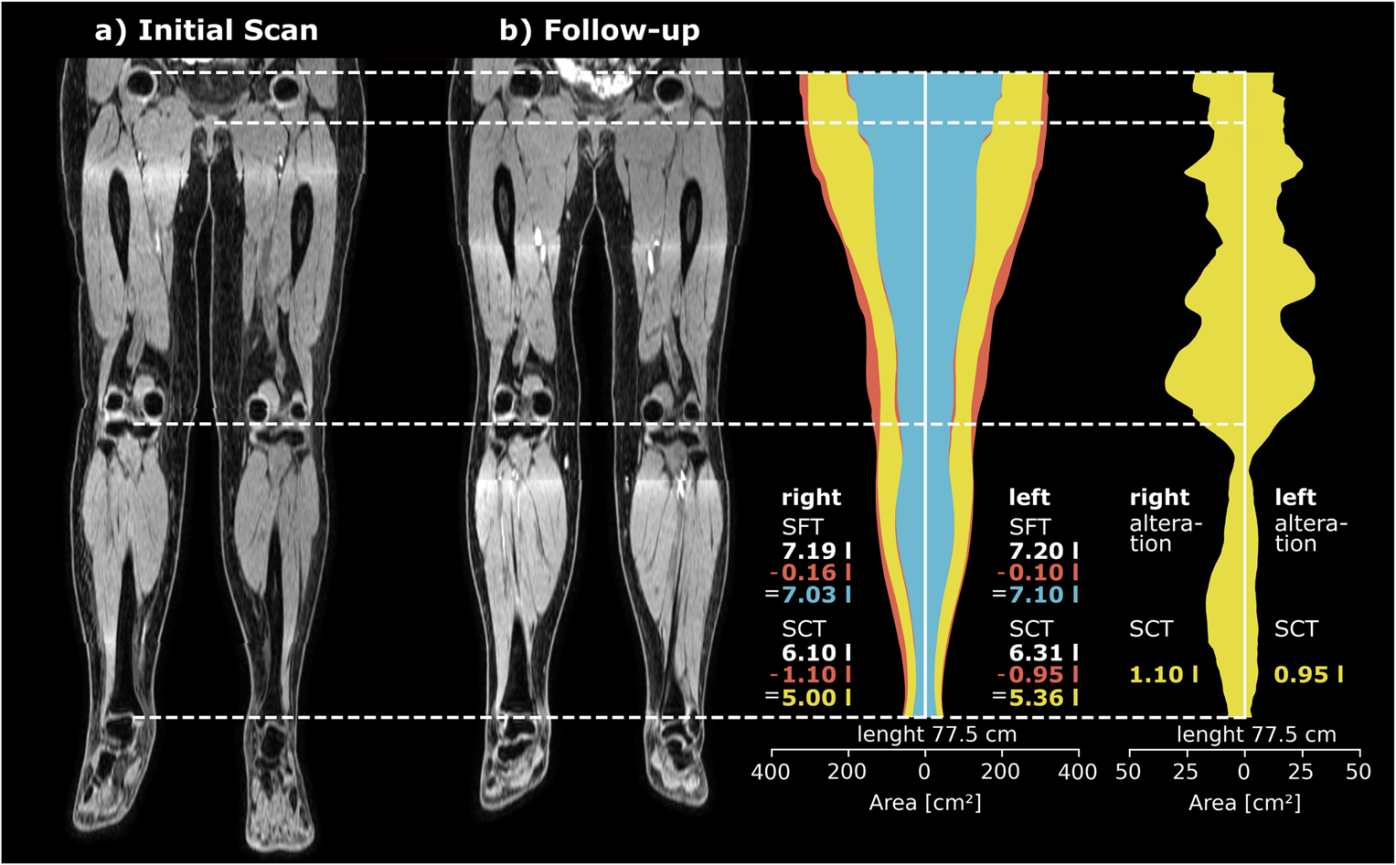
Use case for evaluating success of surgical treatment. The figure illustrates normalized visualizations of a pre-therapeutic and 1-year follow-up scan of a lymphedema patient (female, 55 years old) who received surgical treatment (lympho-venous anastomoses). Cumulative axial tissue areas for the follow-up examination are illustrated, with the distribution of the subfascial tissue (SFT) shown in blue and of the subcutaneous tissue (SCT) in yellow separated for the left and the right leg between the femoral heads and the ankles. The differences in tissue volumes between the initial and the follow-up scan, i.e., tissue portions that have decreased in the course of the treatment, are indicated in red color. Next to the right and left leg, the total volume of SFT and SCT measured at the initial examination is indicated with white font, the total volume of SFT and SCT measured at the follow-up examination is indicated with blue and yellow font, and the decrease in volume is indicated with red font. On the right side of the figure, the alterations in SCT volume between initial and follow-up scan is presented in yellow and in a different scale to highlight where predominantly decrease of tissue volume has occurred during the course of treatment

The trained DL pipeline was applied to different use cases of routine clinical practice to create leg normalized visualizations, which are shown in Figs. 3 and 4.

## Discussion

This work presents a DL method for standardized quantification of subcutaneous and subfascial tissue of the lower extremities in patients with lipedema and lymphedema, which has the potential to provide an in-depth description of shape, volume, and asymmetry.

Modern imaging techniques have become increasingly important in the work-up of patients with suspected lipedema and lymphedema or lymphatic leakages [6, 7, 11, 23, 24]. Especially high-resolution 3D MRL has shown to be useful for planning of new surgical therapeutic options of lymphatic diseases [25] and may also be helpful in treatment follow-up. Usually morphological sequences are part of a MRL protocol and allow for structural assessment of the affected legs. Therefore, it is a logical consequence to apply the capabilities of DL to available morphological 3D imaging to automatically extract information about the exact tissue volumes that might be otherwise unused, which could lead to a more objective assessment of edematous diseases compared to conventional measurements.

Spatial standardization of identified tissues allows comparison between examinations independent of leg length, slice thickness, and position, ultimately allowing comparison of tissue volume distributions between initial and follow-up scans of a patient. To achieve automated standardized analysis, two tasks were solved by utilizing DL, namely tissue segmentation and landmark detection. As a further step towards clinical application, the proposed pipeline in the current study includes a segmentation quality control approach as proposed in a previous work [16]. As an extension to this method that based on the entire 3D volume, we additionally developed a linear regression model trained on each slice of the 3D scan. This allows to assess local quality of the segmentation process and is therefore more sensitive to local effects, e.g., caused by imaging artifacts.

For both the landmark detection and tissue segmentation methods, a 2.5D approach incorporating three slices was chosen. This approach has significantly lower computational costs compared to 3D CNNs, allowing analysis of the high-resolution MRI scans without prior down-sampling, while reducing hardware requirements and time needed for inference. Since excellent results were already observed for the 2.5D approach, the inclusion of more 3D related information through a multi-view approach was not found to be beneficial for the given tasks. Furthermore, the performance and efficiency of different CNN models for landmark detection and tissue segmentation were investigated in this work. The recently released EfficientNet, which showed state-of-the-art performance on the ImageNet dataset at the time of its release while maintaining very efficient computational requirements, was observed also to be high performant and efficient for medical landmark detection and tissue segmentation. Employing efficient models is also of interest for the use of DL in routine clinical practice, as they can reduce costs by further lowering hardware requirements and inference time.

A detailed comparison with previous work on the quantification of tissue volumes for lymphedema assessment in patients with breast cancer using manual landmark definition and non-DL algorithms, as well as previous work on body part detection in medical imaging, can be found in Supplement S6 [8, 26–28].

Our study has several limitations. First, MRI images from routine clinical practice of patients with lipedema and lymphedema, but no patients who are solely obese and have no edematous alterations, were used for the development of the method. However, we assume, although it was not explicitly tested in this study, that the deep learning pipeline also works in purely obese patients without edema, as the high Dice values show that the tissue regions of the patients used for method development, where edema is not present, were also segmented with high precision. Also, data for method development were mainly from female patients. This is due to the fact that lymphedema as well as lipedema occurs predominantly in the female population and data from routine clinical practice was used for this study. However, excellent performance values for landmark detection and tissue segmentation were also observed in male patients of the validation sets. Furthermore, the deep learning method was developed using DIXON water images from a single MRI scanner only. Multi-center trials are warranted to proof the general applicability. The use of the algorithm will be enabled for collaborative multi-center studies on reasonable request (https://qilab.de). Also, at the current stage, there was no further investigation of the segmented tissues with respect to fluid infiltrations, which have implications for treatment strategy of lipedema and lymphedema. In this respect, the presented approach may be used as a basis for further quantitative analyses of tissue properties in future studies, e.g., by multi-parametric imaging. Lastly, the proposed method has not been evaluated for e.g. treatment response assessment in a clinical trial so far. However, we demonstrate potential use cases of the method showing examples for tissue volume assessment, evaluation of asymmetrical tissue proportions, and the evaluation of volume changes after surgical treatment. Future studies should evaluate the clinical value of the method for diagnosis, treatment planning, and treatment monitoring of lipedema and lymphedema against or in compliment of conventional anthropometric measurements.

## Conclusion

This study presents a DL system for standardized and objective analysis of tissue volume, distribution, and symmetry based on MRI in patients with suspected lipedema or lymphedema.

## Supplementary information

ESM 1 (PDF 346 kb)

## Abbreviations

CNN: Convolutional neural network
DL: Deep learning
MRL: MR-lymphangiography
SCT: Subcutaneous tissue
SFT: Subfascial tissue

## Declarations

### Guarantor

The scientific guarantor of this publication is PD Dr. med. Claus C. Pieper.

### Statistics and biometry

No complex statistical methods were necessary for this paper.

### Methodology

• retrospective

• experimental study

• performed at one institution
