# Supplementary material for: Deep learning for standardized, MRI-based quantification of subcutaneous and subfascial tissue volume for patients with lipedema and lymphedema
Source: Eur Radiol. 2022 Aug 17;33(2):884–92. doi: 10.1007/s00330-022-09047-0 (PMC9889496; doi:10.1007/s00330-022-09047-0)
Supplement: Supplementary file 1 — (PDF 346 kb) [file 330_2022_9047_MOESM1_ESM.pdf]

Supplement

S1 Detailed imaging parameters and image pre-processing

MRL was performed on a 1.5 T MR-system (Ingenia; Philips Healthcare) with the patients in supine position and outstretched legs. A morphological axial 3D T1-weighted DIXON sequence was part of the clinical examination protocol in all patients for anatomical evaluation of the legs (TR 5.8ms, TE 1.8ms and 4ms, flip angle 15°, field of view 450x345mm, slice thickness 5mm, spacing between overlapping reconstructed slices 2.5mm, in-plane resolution 1mm, acquisition time per stack 65s). Four stacks were necessary to image the entire legs in all patients.

Before training, the image values of the scans were z-score normalized. Therefore a body mask was created by filtering the image with a minimum filter of kernel edge length 3 and then selecting all voxels with image values above the global minimum. Finally, the largest connected segment of this mask was defined as the body mask. The image values were then transformed to have mean 0 and standard deviation 1 within the mask.

The axial images were transformed to the median matrix size of the dataset (400) by applying cubic interpolation. In order to remove regions containing only image background, the matrices were then cropped to a matrix size of 330 in anterior-posterior direction.

S2 Detailed information on the annotation process

For ground truth generation of the SCT and SFT segmentation, semi-manual tools were used in Slicer 3D by a research assistant (S.N. with 3 years of experience in medical image segmentation). For semi-manual segmentation the segmentation editor module of 3D Slicer was used [1]. After annotating two and again after annotating six patients semi-manually, a convolutional neural network was trained with these initial segmentations to support further annotations. All segmentations were optimized using semi-manual tools and were finally approved by a board-certified radiologist (C.C.P. with 10 years of experience in lymphatic imaging). Landmark definitions of ankles, knees, symphysis and femoral heads were also performed in Slicer 3D by the research assistant and reviewed by the certified radiologist.

S3 Detailed information on the model architectures

In this study different versions of a modern implementation of the established ResNet, as well as different versions of the recently introduced EfficientNets were implemented as encoders for the convolutional neural networks (CNN) for landmark detection and tissue segmentation [2, 3]. The implementation of the PyTorch based API FastAi (version 2.3.0, https://github.com/fastai/fastai) was used for the ResNets. The implementation of the PyTorch Image Model repository (version 0.4.9, https://github.com/rwightman/pytorch-image-models) by Ross Wightman was used for the EfficientNets [4, 5]. For tissue segmentation, a U-Net version of all ResNets and EfficientNets was generated using FastAi's DynamicUnet class, which builds a decoder on top of the architectures using PixelShuffle ICNR upsampling and two convolutional layers for each resolution level [6]. The Competitive Dense Fully-Convolutional Network (CDFNet) was obtained also from Github (https://github.com/reuter-lab/FatSegNet_pytorch) and compared to the ResNet and EfficientNet models.

S4 Hyperparameters used while training

Model training was performed in PyTorch and with the application programming interface FastAi, using mixed precision training [4]. The landmark detection methods were trained on a Nvidia RTX 3090 Graphics Processing Unit (GPU) and the tissue segmentation methods were trained on an Nvidia Titan RTX GPU.

Leg Model Regression: The Adam optimization algorithm, a batch size of 64, mean-squared-error loss function, a weight decay of 1e-6, and early stopping was used for training. The learning rate and momentum followed a cyclical scheme during 120 epochs of training, according to the one cycle learning rate policy [7]. With this scheme, the learning rate was increased to a maximum value of 0.01 until 30% of the training epochs, starting from the maximum value divided by 25. Then the learning rate was dropped towards zero by cosine annealing until the last epoch. The momentum was dropped from 0.95 to 0.85 and then raised back to 0.95. All 2D images were augmented by affine transformations with a probability of 75% by rotating by -10° to 10° and scaling by 0% to 10% while assembling the batches for training the Leg Model Regression method.

Keypoint Detection: The Adam optimization algorithm, a batch size of 64, mean-squared-error loss function, a weight decay of 1e-6, and early stopping were also used for training the keypoint detection. The same one cycle learning rate policy was applied, however with a total of 1500 epochs. While assembling the batches for training, the images were also augmented with a probability of 75% by rotating by -10° to 10°, however scaling was 0% to 50% with cropping and vertical flipping was applied.

Tissue Segmentation: The Adam optimization algorithm, a batch size of 16, cross-entropy loss function, a weight decay of 1e-6, and early stopping was used for training. The same one cycle learning rate policy was applied, however with a total of 20 epochs and a maximum learning rate of 0.001.

S5 Detailed information on model selection

In order to select an optimal model for the tasks of this study, all implemented CNNs were compared based on their performance on validation data and their efficiency as measured by the number of floating point operations required and the number of trainable parameters (Figure S6). The number of required floating point operations and the number of trainable parameters of the models were determined using the package fvcore (version 0.1.5, https://github.com/facebookresearch/fvcore). The mean validation performance of the 5-fold cross-validated landmark detection models and the validation performance of the first split of the tissue segmentation were used for evaluation. Results are shown in Figure S5. All tested models showed a high performance for the tasks of this study. The EfficientNet models were slightly better for both landmark detection methods, with lower numbers of trainable parameters and floating point operations. In the segmentation of subcutaneous tissues, all models studied showed a similar high Dice score. The CDFNet was found to have the fewest trainable parameters, yet the most required floating point operations. This could be related to the none-trainable operations of the competitive feature selection, which compares each value within two equally sized feature maps and selects the larger of the two values in each case. Due to the low number of trainable parameters and required floating point operations, as well as the high

performance in all tasks, the EfficientNet-B1 was chosen for both the landmark detection methods and the tissue segmentation.

S6 Detailed comparisons to other works

A previous paper investigated the clinical utility of quantifying tissue volumes for the assessment of lymphedema in patients with breast cancer [8]. For tissue segmentation and standardized visualization of the upper extremities, the authors applied a k-means algorithm and manual definition of landmarks. In the current study, and in contrast to this previous work, the tissue segmentation and landmark detection was performed automatically by DL and all methods were validated by five-fold cross-validation and tested on hold-out test data. The use of DL also enables the implementation of quality control methods based on the entropy of the predicted probability [9].

Other works on body part recognition in medical imaging feature also different approaches. An earlier work realizes the recognition of human body parts by dividing the body into fixed discrete classes over hard boundaries in the foot-head direction, and then training a CNN to assign all slices of a scan to one of twelve body part classes [10]. This approach has drawbacks, as subsequent further partitioning between sub-areas is not possible and as uncertainties can occur in areas where abrupt partitioning between the body part classes are applied. Therefore, later work has described body parts with a model using continuous values [11]. In another work, the continuous values that represent the body model are learned by the CNN in an unsupervised training by considering the order and distance between axial layers [12]. This approach has the benefit that no manually defined landmarks are required for training. The output of this unsupervised trained CNN yields a height ranking of the slices, and the anatomical regions have similar output values in all patients. However, since no manually defined values are used for training, there is initially no information on how to map the numbers predicted by the CNN to a specific landmark. This means, that manually defined landmarks are still required for the mapping of the output of the CNN to the searched anatomical landmarks. For this reason, and due to the fact that such an unsupervised learning approach requires a much larger amount of data, we manually defined the landmarks in our cohort and used this information to build the leg model, which led to excellent regression results. Furthermore, the manual definition of the landmarks combined with the creation of reconstructed, coronal slices further improved the performance by developing a second method for identifying the landmarks, namely keypoint detection that is commonly applied for the identification of human pose in natural images [13].

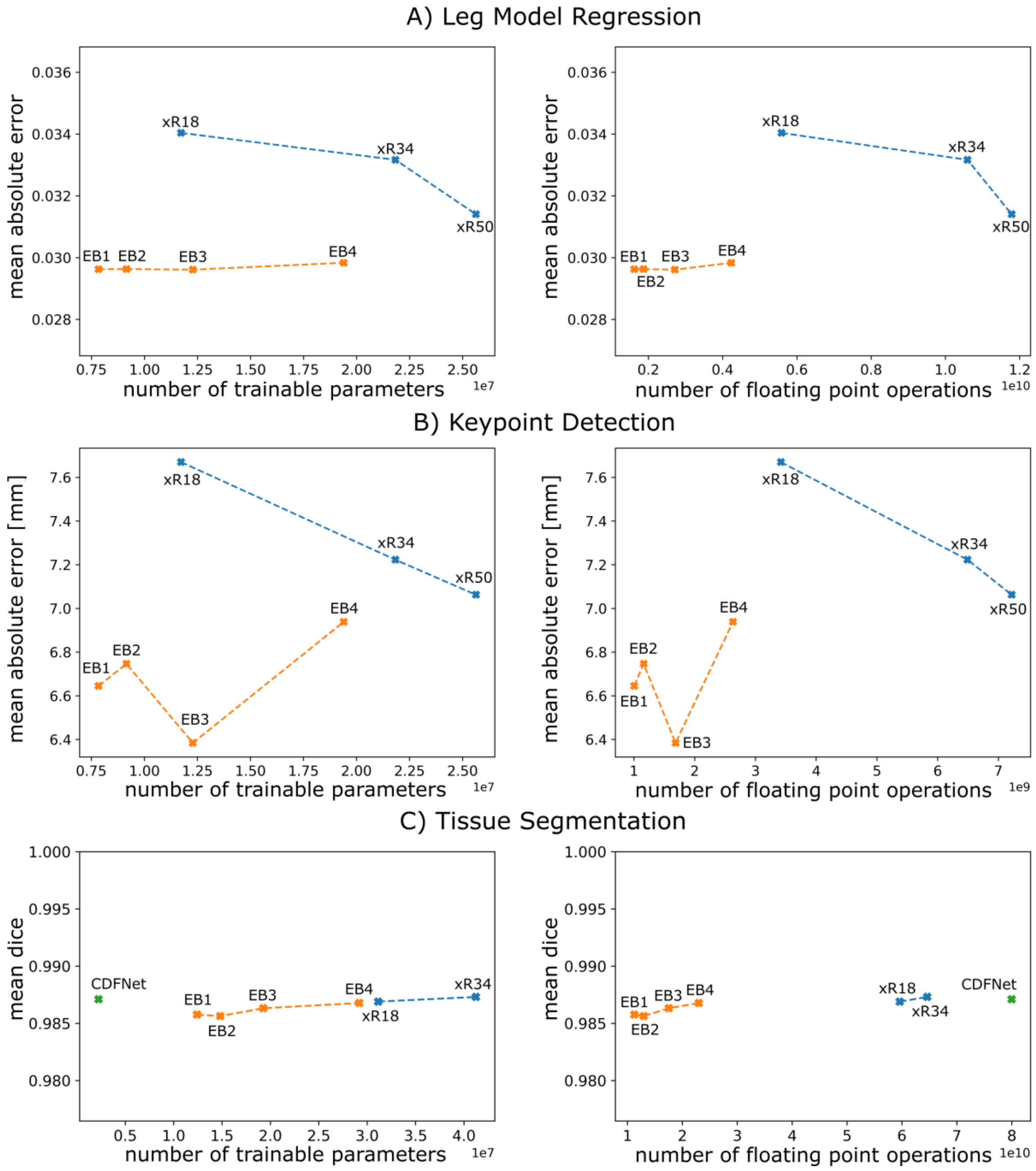

Fig S5: Performance on the validation data of all models examined versus the number of trainable parameters and floating point operations required.

References

1. Fedorov A, Beichel R, Kalpathy-Cramer J et al. (2012) 3D Slicer as an image computing platform for the Quantitative Imaging Network. Magn Reson Imaging, 30(9):1323-1341

2. He T, Zhang Z, Zhang H, Zhang Z, Xie J, Li M (2019) Bag of tricks for image classification with convolutional neural networks. In Proceedings of the IEEE/CVF Conference on Computer Vision and Pattern Recognition, pp. 558-567

3. Tan M & Le Q (2019) Efficientnet: Rethinking model scaling for convolutional neural networks. In International Conference on Machine Learning, pp. 6105-6114

4. Howard J, Gugger S (2020) Fastai: A Layered API for Deep Learning. Information 11(2):108

5. Wightman R (2019) Pytorch Image Models. GitHub repository. DOI: 10.5281/zenodo.4414861, Available via https://github.com/rwightman/pytorch-image-models

6. Shi W, Caballero J, Huszár F et al. (2016) Real-Time Single Image and Video Super-Resolution Using an Efficient Sub-Pixel Convolutional Neural Network. Proceedings of the IEEE conference on computer vision and pattern recognition, pp. 1874-1883

7. Smith LN (2018) A disciplined approach to neural network hyper-parameters: Part 1 -- learning rate, batch size, momentum, and weight decay. arXiv:180309820

8. Borri M, Gordon KD, Hughes JC et al. (2017) Magnetic resonance imaging–based assessment of breast cancer–related lymphoedema tissue composition. Invest Radiol 52(9):554

9. Mehrtash A, Wells WM, Tempany CM, Abolmaesumi P, Kapur T (2020) Confidence calibration and predictive uncertainty estimation for deep medical image segmentation. IEEE Trans Med Imag 39:3868–3878

10. Yan Z, Zhan Y, Peng Z et al. (2016) Multi-instance deep learning: Discover discriminative local anatomies for bodypart recognition. IEEE Trans Med Imaging 35(5):1332-1343.

11. Zhang P, Wang F, Zheng Y (2017). Self supervised deep representation learning for fine-grained body part recognition. In IEEE 14th International Symposium on Biomedical Imaging, pp. 578-582

12. Yan K, Lu L, Summers RM (2018) Unsupervised body part regression via spatially self-ordering convolutional neural networks. In 2018 IEEE 15th International Symposium on Biomedical Imaging, pp. 1022-1025

13. Dang Q, Yin J, Wang B, Zheng W (2019) Deep learning based 2d human pose estimation: A survey. Tsinghua Sci Technol 24(6):663-676
